# Characteristics, Mechanism and Criterion of Channel Segregation in NbTi Alloy via Numerical Simulations and Experimental Characterizations

**DOI:** 10.3390/ma14040796

**Published:** 2021-02-08

**Authors:** Baohui Zhu, Zhenzhen Chen, Yanfei Cao, Yanchang Liu, Xiuhong Kang, Yun Chen, Hongwei Liu, Paixian Fu, Yikun Luan, Dianzhong Li

**Affiliations:** 1 State Key Laboratory of Special Rare Metal Materials, Northwest Rare Metal Materials Research Institute Ningxia Co., Ltd., Shizuishan 753000, China; zbhwel@163.com (B.Z.); yanchang.liu@163.com (Y.L.); 2 Shenyang National Laboratory for Materials Science, Institute of Metal Research, Chinese Academy of Sciences, Shenyang 110016, China; zhzhchen19s@imr.ac.cn (Z.C.); xhkang@imr.ac.cn (X.K.); chenyun@imr.ac.cn (Y.C.); hwliu@imr.ac.cn (H.L.); pxfu@imr.ac.cn (P.F.); ykluan@imr.ac.cn (Y.L.); 3 School of Materials Science and Engineering, University of Science and Technology of China, Hefei 230026, China

**Keywords:** NbTi alloy, channel segregation, simulations, experimental characterizations, criterion

## Abstract

Channel segregation (CS) is the most typical defect during solidification of NbTi alloy. Based on numerical simulation and experimental characterizations, we deeply elucidated its characteristics, formation mechanism, effecting factor and prediction criterion. According to acid etching, industrial X-ray transmission imaging, 3D X-ray microtomography and chemical analysis, it was found that in a casing ingot, by He cooling, finer grain size, weaker segregation and slighter CS can be obtained compared with air-cooled ingot. The simulation results of macrosegregation show that CS is caused by the strong natural convection in the mushy zone triggered by the thermo-solutal gradient. Its formation can be divided into two stages including channel initiation and growth. In addition, due to the stronger cooling effect of the He treatment, the interdendritic flow velocity becomes smaller, consequently lowering the positive segregation and CS and improving the global homogenization of the final ingot. Finally, to predict the formation of CS, the Rayleigh number model was proposed and its critical value was found to be 15 in NbTi alloy for the first time. When it is lower than the threshold, CS disappears. It provides an effective tool to evaluate and optimize the solidification parameters to fabricate the homogenized NbTi ingot in engineering practice.

## 1. Introduction

As the common material of low-temperature superconductors, NbTi alloy has been widely used in high-energy physics, controlled thermonuclear fusion, energy storage and magnetic levitation considering its superior machinability and high critical current density [1,2,3]. To achieve the low magnetic hysteresis loss and residual resistivity ratio, the high compositional homogenization and low segregation index are strictly required in the casting ingot and product. Owing to the large density difference between Nb and Ti, the severe Ti segregation occurs during solidification of ingots, which always causes the breakage of NbTi superconducting wire [4,5]. 

In NbTi alloy, channel segregation (CS, namely freckle, chimney) is the most common type of macrosegregation during solidification. It cannot be eliminated by the subsequent deforming and heat treatment processes considering its large scale of width in ~mm and length in ~cm. More importantly, due to the thermal-solutal distribution feature of the ingot, CS is usually located in the 1/3–2/3 radius and, hence, it also cannot be cut off by punching or peeling. 

Actually, CS also exists in the common structural materials of aluminum alloy, Ni-base superalloys, and steels and functional materials such as SnPb, SnBi and GaIn [6,7,8,9,10,11,12,13,14,15,16]. According to the macrosegregation theory, CS is caused by the redistribution of solute and natural convection during solidification [17,18,19,20,21,22,23]. With the development of solidification, the redistribution phenomenon occurs due to the difference of the solubility of solutes in solid and liquid phases. When solute continuously segregates and enriches in front of solidification, the interdendritic liquid flows upwards considering the huge density inversion between the mushy zone and bulk liquid, and even becomes instable. Under the interaction between flow and solidification, the sequential solidification is destroyed and solidification is restricted, which initiates the CS. Such mode has been verified in the above-mentioned alloys, but the formation mechanism and evolution has been not reported in NbTi alloy. 

On the other hand, the detailed characteristics and features of CS in NbTi alloy are also unclear currently. In industry, the size and distribution of CS are usually only observed by industrial-level X-ray transmission. However, its resolution is too low to capture the details of CS formation. Moreover, the quantitative information and segregation extent cannot be given by such qualitative characterization. Hence, here its formation details will be revealed by combining multiscale experiments such as grain size statistics, 3D morphology observation and chemical analysis and numerical simulations. 

In this study, based on numerical simulations and experimental characterizations, the characteristics, evolution mechanism and effecting factor of CS are investigated in detail in NbTi alloy. In addition, the prediction criterion of CS is proposed to provide the basis to optimize the solidification process and to produce a homogenized NbTi ingot.

## 2. Materials and Experimental Methods 

### 2.1. Ingot Production and Industrial X-ray Transmission Observation 

Two Nb-47 wt.% Ti ingots with Φ530 mm × 2700 mm were produced by He and air cooling between a copper mold and melt, respectively. Because of the low temperature of He, its cooling effect is strengthened compared with the normal air-cooled ingot. After the complete solidification, it was dissected along the longitudinal center plane. A test piece of dimensions 250 mm × 170 mm × 4 mm was cut from the top surface to perform the industrial X-ray transmission observation. The Paxscan 2530C X-ray apparatus (Varian Medical Systems, Palo Alto, CA, USA) was used with a current of 1 mA and a voltage of 128 kV. Its resolution is 139 μm, and the final image includes a total of 256 frames. The imaging is based on the density difference of the inner materials and they present the distinct X-ray absorption capacities, consequently causing the different signal strength in the detector. Owing to Ti enrichment in CS, its density is lighter and its X-ray absorption capacity of is weaker than the normal zone, which leads to the blacker imaging of banded CS. 

### 2.2. Acid Etching and Grain Size Distribution 

To obtain the morphology and size of the grains in two ingots, the above pieces were etched by the mixed acid with H_2_SO_4_:HF:HNO_3_ of 5:1:2 in volume. The etching time was about 55 s and then the surface was cleaned and dried. Due to the distinct corrosion resistance between the grain boundary and interior, the grain morphology can be observed. By such a macro etching technique, the effect of the different cooling methods on the final grain distribution in the ingot can be revealed. Quantitatively, the statistic software MIPAR v3.3.4 was used to calculate the numerous grains. By using the cutting line method, the shape parameters and dimension of each grain can be obtained. Meanwhile, the statistic grain size is applied as the input parameter in the subsequent macrosegregation simulations.

### 2.3. Chemical Analysis and Segregation Determination 

To illustrate quantitatively the effect of He cooling on the final Ti segregation, five samples were drilled uniformly at the half height of the piece obtained in Section 2.1. The distance between the neighboring points is 50 mm. Then, the sample was dissolved in acid and the solute content was detected by inductively coupled plasma-atomic emission spectrometry (ICP-AES) with an iCAP6300 (Thermo Fisher Scientific, Waltham, MA, USA). 

### 2.4. 3D Microtomography

To reveal the 3D distribution of CS in two ingots, cylinder samples of Φ2 mm × 28 mm were cut in the center of the test piece. After being ground by the abrasive paper and ultrasonic cleaning, sample was put on the stage of an Xradia Versa XRM-500 (Carl Zeiss AG, Shanghai, China) [24]. Its voltage and resolution are 140 kV and 30 μm, respectively. With rotation of 360°, total 1600 2D projection images can be obtained and the exposure time was 4 s. Then, these 2D images can be reconstructed into the 3D image after Foulier filtering by the back projection algorithm. Finally, via the post processing, visualization and computing using Avizo fire 7.1 software, the 3D inner structure of CS can be reproduced. 

## 3. Experimental Results

**Figure 1:**
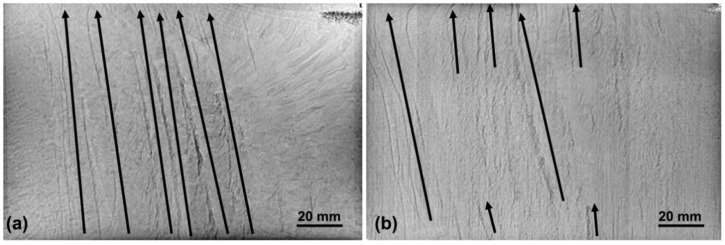
Macrostructure and channel segregation distributions in the top slice of Air- (**a**) and He-cooled (**b**) ingots of NbTi alloy. Channel segregation (CS) is marked by the black arrow.

The distributions of CS near the top surface in He- and air-cooled NbTi ingots revealed by the industrial X-ray detector are shown in Figure 1. Different from the dotted “freckle” in the horizontal section, it appears to be band or channel in the current longitudinal section as marked by the black arrows. In the normal air-cooled ingot, CS is mainly distributed in 1/4–3/4 radius, and most channels can run through the whole height of the test piece. In contrast, in the He-cooled ingot, the formation positions of CS are generally the same with the air-cooled ingot, but the color of CS becomes lighter. The more severe Ti segregation is, the darker CS becomes. In addition, after He-cooled treatment, the distance between neighboring channels is larger, and its length is shorter and hence it cannot span the whole section in height. 

**Figure 2:**
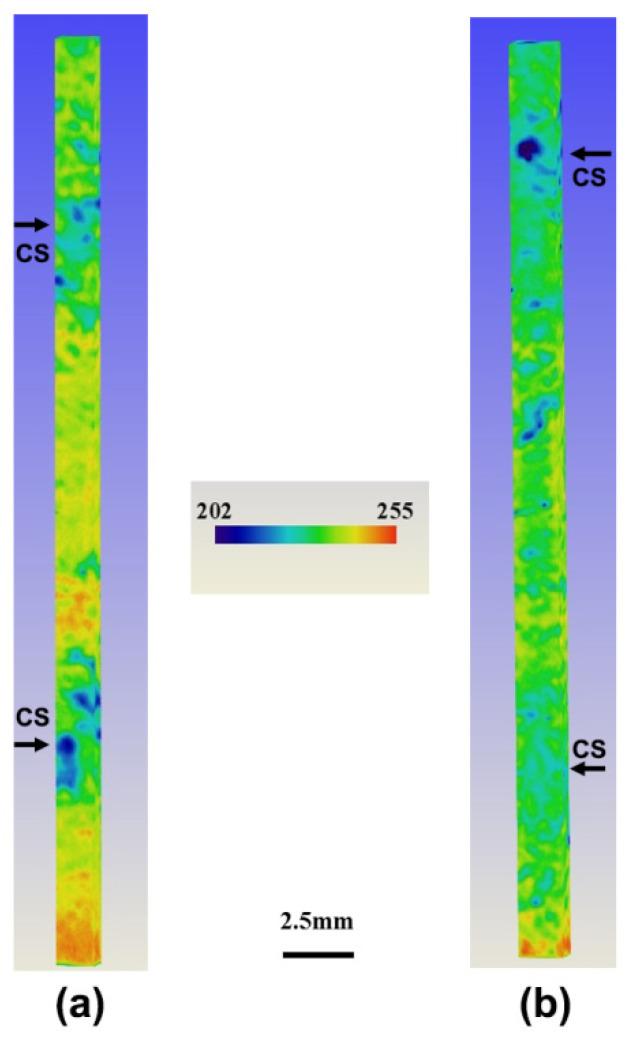
The three-dimensional distribution of grey scale and CS in Air- (**a**) and He-cooled (**b**) NbTi ingots by 3D microtomography. The sample of Φ2 mm × 28 mm was obtained in the center of the test piece, and the reconstructed zone is 1.875 × 1.875 × 25 mm^3^. The observed two channels (blue color) are marked by the black arrows.

Though Figure 1 reproduces the overall view of CS in the whole section of the ingot, the local detail and 3D morphology of CS cannot be revealed clearly considering its lower resolution of 139 μm. Furthermore, Figure 2 presents the 3D distribution of grey scale and CS via 3D microtomography. Owing to the distinct absorption capacity between CS and normal zones, the grey scale indicates the difference of Ti content in the observation zones, and, correspondingly, the CS formation or lack of formation can be also illustrated. Here, there are two channels marked by the black arrows in the observed zone in both ingots. In the interior of each CS, the grey scale is not the same, indicating the difference of Ti content during the growth of the channel, whose detail cannot be found by the industrial X-ray detector due to its low resolution. In addition, it can be found that there is the extremely nonuniform distribution of Ti content in the air-cooled ingot, and the distance between the neighboring channels of the detected area is about 13 mm. Compared to the normal zone, the grey scale in the CS is obviously lower, indicating the severe segregation of Ti in the air-cooled ingot. However, in the He-cooled ingot, the nonuniformity of grey scale and Ti segregation is significantly reduced, and CS is also obviously slighter. It agrees well with the industrial X-ray transmission result, and He cooling can promote the homogenization of Ti content in NbTi alloy. It should be noted that, the discrepancy of CS features via the two distinct transmission techniques is caused by the heterogeneous distribution of the channel in the three-dimensional spacing and the interaction between 2D slices with different depths, consequently, to some extent, lowering the severity of CS in 3D reconstruction. 

**Figure 3:**
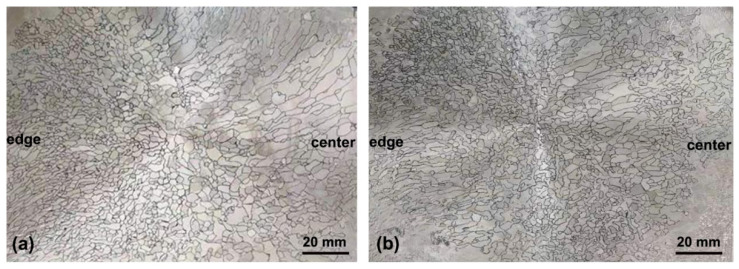
Grain morphology and distribution in the top slice of Air- (**a**) and He-cooled (**b**) ingots of NbTi alloy.

**Figure 4:**
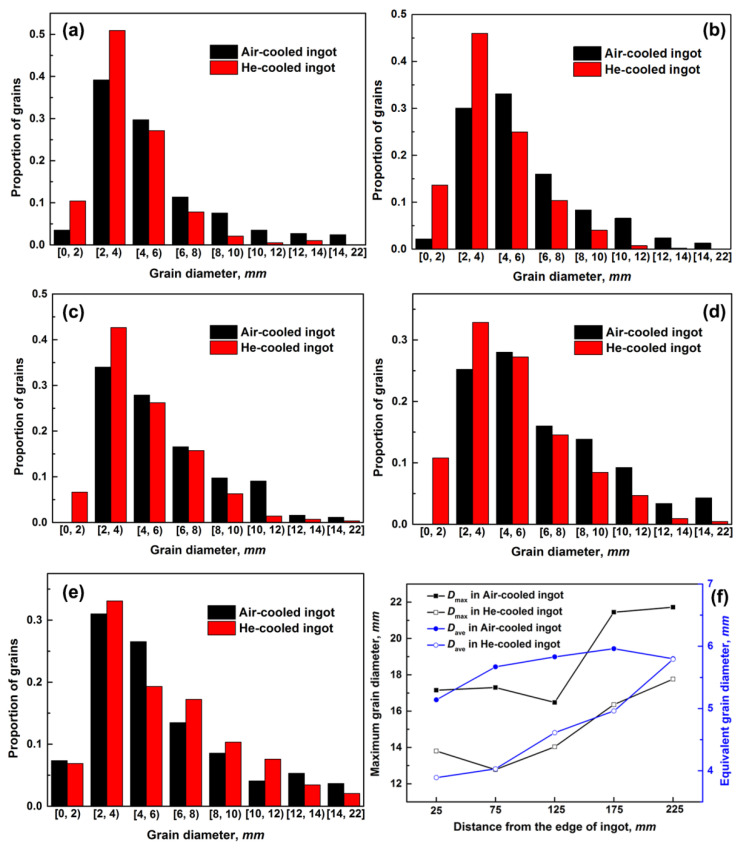
The statistic grain size distributions from the edge to the center of He- and Air-cooled NbTi ingots. (**a**–**e**) With the distances of 0–50, 50–100, 100–150, 150–200 and 200–250 mm, respectively; (**f**) the comparison of maximum (*D*_max_) and average (*D*_ave_) grain sizes in different distances of two ingots.

The final grain morphology and size are revealed in Figure 3. After He cooling, grains become more equiaxed and their dimension is also smaller. The difference of the oriental grain-size distribution from edge to center of the ingot is compared in Figure 4. In both ingots, the grain sizes are mainly distributed from 2 mm to 8 mm (Figure 4a–e). With the increase in distance from the ingot edge, the grain size becomes larger. At the edge of the air-cooled ingot, the maximum (*D*_max_) and average (*D*_ave_) grain sizes are 17.15 mm and 5.14 mm, respectively, and they increase to 21.72 mm and 5.8 mm in the center, respectively. As a comparison, grain size shifts toward the left direction in the He-cooled ingot, indicating the increasing number of small grains. The proportions of the small grains (<5 mm) are 66% and 72%, respectively, in air- and He- cooled ingots. Specially, the ultra-large grains obviously decrease in the He-cooled ingot, and *D*_max_ in the edge and center are lowered to 13.8 mm and 17.16 mm, respectively. 

**Figure 5:**
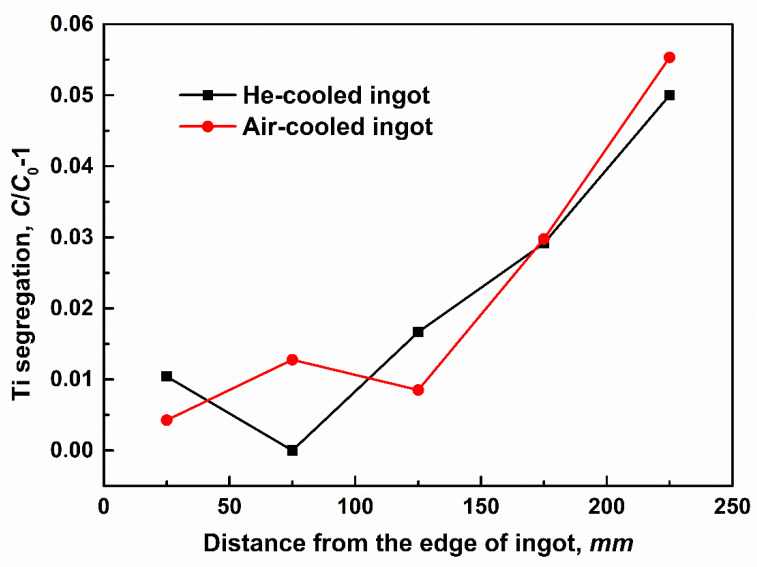
The comparison of Ti segregation from the edge to the center of He-and Air-cooled NbTi ingots.

Quantitatively, the relative segregation of Ti, namely *C*/*C*_0_ − 1, is depicted in Figure 5. Because the samples were obtained from the top surface of the ingot, all the segregations are determined to be positive. In the late solidification, the accumulation phenomenon of solute is rather obvious due to its longer local solidification time. Thus, with the increase in distance from the ingot edge, the Ti segregation becomes more severe considering the solute enrichment caused by the interdendritic convection. It should be noted that, although there is a similar evolution tendency of Ti content in both ingots, quantitatively the maximum and minimum values of Ti segregation are lowered to 0.55 and 0, respectively, in the He-cooled ingot compared to the larger segregation of 0.055 and 0.0043 in the air-cooled ingot. In addition, besides the maximum and minimum segregations, the global macrosegregation extent of *GM*, which indicates the severity of compositional fluctuation in the whole section, is also a key indicator and its expression is below,

(1) $$GM=\frac{1}{n}\sum_{i=1}^{n}(\frac{C_{i}}{C_{0}}-1{)}^{2},$$

where *n* is the sample number, *C* and *C*_0_ are the detected and normal compositions of Ti, respectively. The larger *GM* is, the more severe global segregation becomes. Calculations show that *GM* is 0.0042 and 0.0037, respectively, in the air- and He-cooled ingots, which again proves the positive effect of He-cooling treatment on the final compositional homogenization in NbTi alloy. 

**Figure 6:**
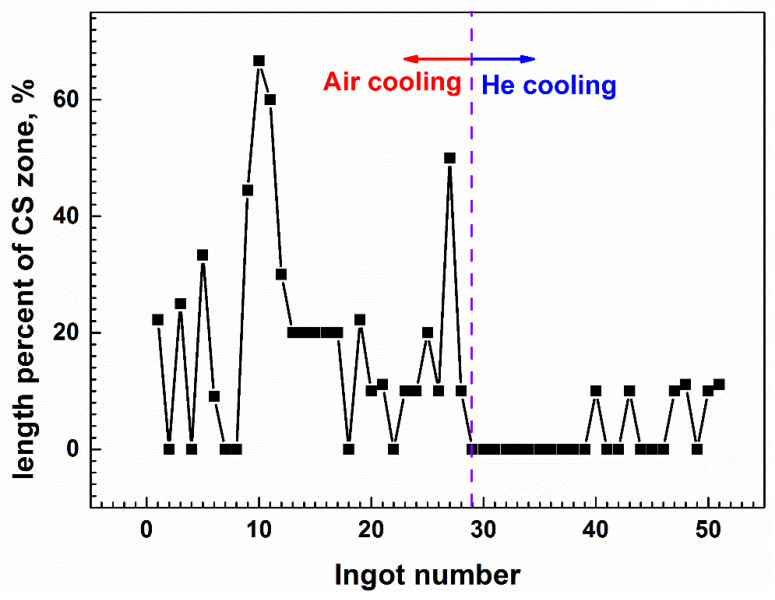
Statistics of the length percentage of the CS zone in the ingot body in Air-and He-cooled ingots.

By comparing these multiscale characteristics of macrosegregation, especially CS in two different cooling processes, it can be concluded that, with the increase in the cooling capacity in the He-cooled ingot, the solidification speed becomes faster during solidification of liquid alloy. As a result, the ejection of solute into the interval of dendrites is suppressed, and the microsegregation becomes slighter. In addition, the higher cooling rate can refine the grains, in which case the permeability in the mushy zone decreases sharply and the thermo-solutal convection strength becomes weaker, consequently lowering the final Ti segregation. When natural convection is suppressed, the risk of solidification destabilization triggered by thermo-solutal fluctuation is lower, and the sequential solidification mode can last, and its ignition of CS becomes more difficult. Even though CS ignition can occur, its growth rate is also restricted, and hence the final CS appears to be less, the shorter length and the lighter color shown in Figure 1. Actually, to sufficiently validate the positive effect of He cooling on the final compositional homogenization, CS severity in more 51 Nb-47 wt.% Ti ingots (Φ530 mm × 1700–2700 mm) was statistically detected by scanning the whole ingot body along the longitudinal direction and the grassy-shape wave received in an ultrasonic fault detector. The length ratio between the CS zone and the whole body is introduced in Figure 6. It shows that, after He cooling, the average and maximum length percentages of the CS zone are lowered to 2.83% and 11.11% from 18.76% and 66.67% by the air cooling treatment, respectively. 

## 4. Simulation Method and Model

To reveal the formation mechanism and evolution detail of CS in NbTi alloy, a series of macrosegregation simulations were further carried out by considering the thermal and solutal effects during solidification. In the model, the local thermo-solutal balance is assumed to be met at the solid/liquid interface. The model details can be referred to in our previous publication [25], and here only the conservations of solute, energy and momentum are listed below.

The momentum equation in *x* direction:

(2) $$\frac{\partial \left(\rho u\right)}{\partial t}+\nabla \cdot \left(\rho \vec{U}u\right)=-\frac{\partial P}{\partial x}-Ku+\nabla \cdot \left(\mu_{l}\nabla u\right),$$

The momentum equation in y direction:

(3) $$\frac{\partial \left(\rho w\right)}{\partial t}+\nabla \cdot \left(\rho \vec{U}w\right)=-\frac{\partial P}{\partial z}-Kw+\nabla \cdot \left(\mu_{l}\nabla w\right)+\rho g\left[\beta_{T}\left(T-T_{ref}\right)+\beta_{C}\left(C_{l}-C_{ref}\right)\right],$$

The energy equation:

(4) $$\frac{\partial \left[\rho H\right]}{\partial t}+\nabla \cdot \left(\rho c_{p}\vec{U}T+\rho \vec{U}\Delta H\right)=\nabla \cdot \left(\lambda \nabla T\right),$$

The solute equation:

(5) $$\frac{\partial [\rho C]}{\partial t}+\nabla \cdot \left(\vec{U}C_{l}\right)=0,$$

In the model, to calculate the solid fraction *f*_s_ during solidification accurately, the following quadratic equation is built by combining the energy and solute relationship.

(6) $$\begin{array}{l} A\cdot {\left(f_{s}^{t}\right)}^{2}-B\cdot f_{s}^{t}+E=0\\ where\\ A=\left(1-k\right)\cdot \rho \cdot \Delta H\\ B=\rho \Delta H(k+kf_{s}^{t-\Delta t}-2)+(k-1)(\rho c_{p}T_{M}-{\left[\rho H\right]}^{t})\\ E=(\rho c_{p}T_{M}-{\left[\rho H\right]}^{t}+\rho \Delta H)(1-kf_{s}^{t-\Delta t})-\rho c_{p}m_{l}C_{l}^{t-\Delta t}(1-f_{s}^{t-\Delta t}) \end{array},$$

**Figure 7:**
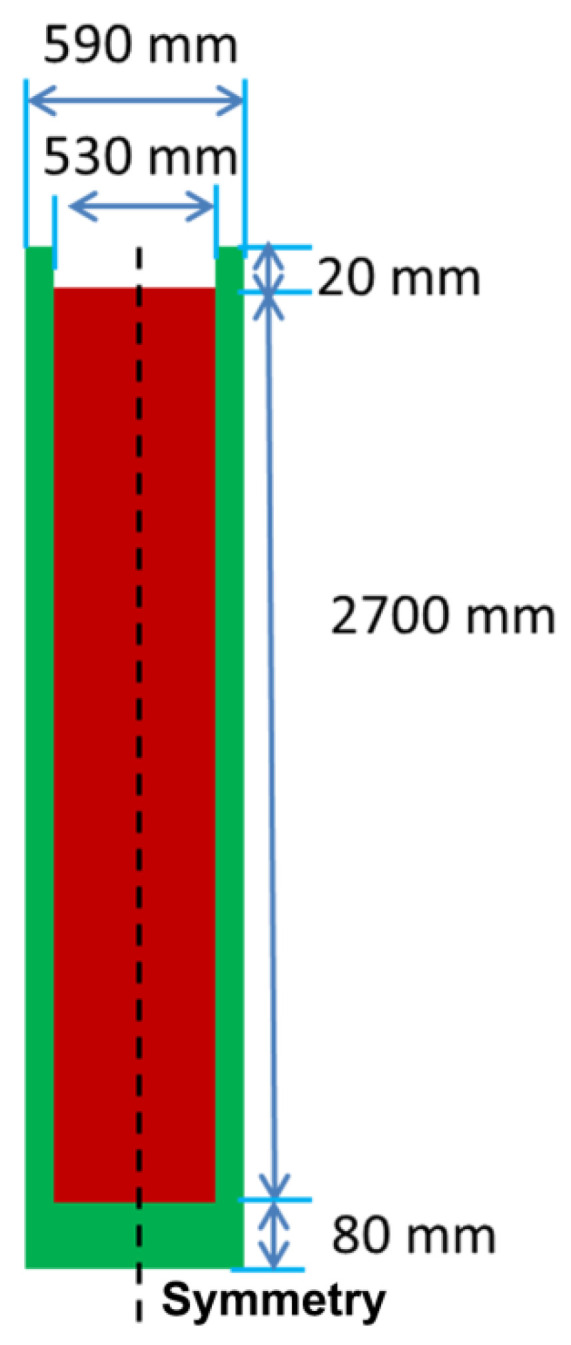
The geometry and dimensions of the simulated NbTi ingot.

The finite difference method is applied to solve the above conservation equations, and in the momentum equation, the correction between velocity and pressure is realized via the SOLA algorithm. In the current simulation cases, the experimental ingot of Nb-47 wt.% Ti with Φ530 mm × 2700 mm was simulated as shown in Figure 7, and its grid size was 3 mm × 3 mm. Considering the geometrical symmetry and computational efficiency, only the left part of the ingot was calculated. Based on the experimental result of grain size, the secondary dendritic arm spacing *d*_s_ was selected as 2000 μm. The thermo–physical parameters and boundary conditions are listed in Table 1 and Table 2, respectively.

## 5. Simulation Results and Discussion

### 5.1. Formation Mechanism of CS in NbTi Alloy 

**Figure 8:**
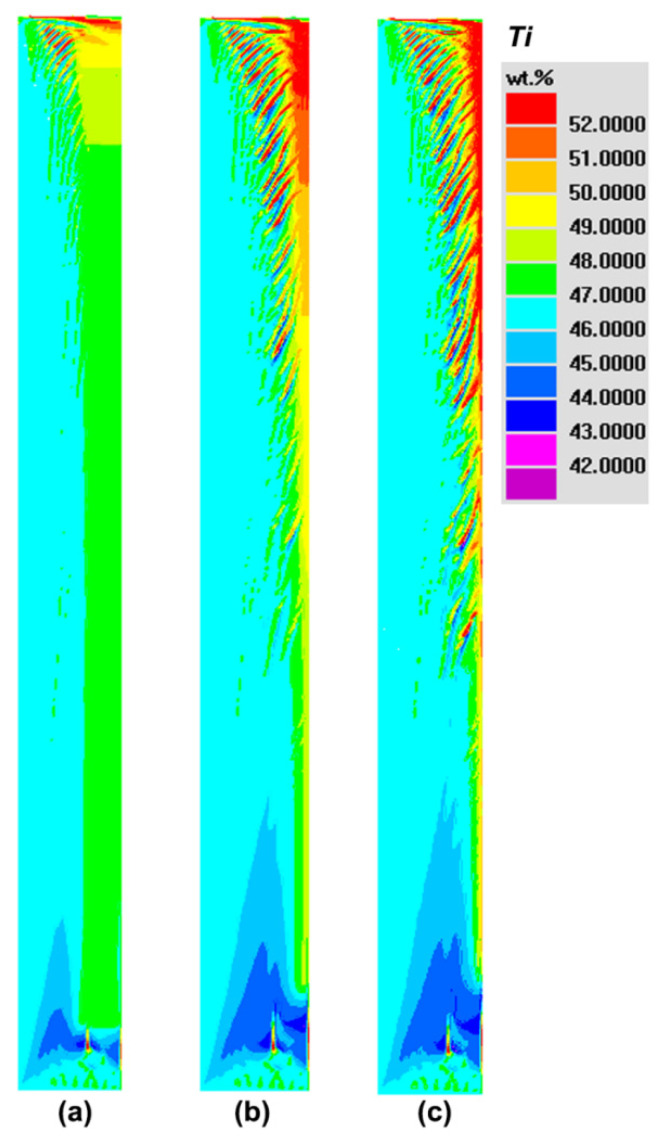
The macrosegregation distribution and evolution of Ti in the NbTi ingot. (**a**) 1000 s; (**b**) 2700 s; (**c**) 11,000 s. At 2700 s, a solidification front arrives appropriately at the half of the ingot. At 11000s, the solidification of the ingot is complete.

Figure 8 reveals the evolution process of Ti segregation during solidification. Simulations show that, with the increase in solidification time, the segregation phenomenon becomes more severe. After the final solidification, the positive segregation in the top, the negative segregation in the bottom and channel segregation in both sides of the ingot body are reproduced, which are totally consistent with the typical types of macrosegregation in the casing ingot. Hence, the current model and simulations can be used to elucidate the formation mechanism and characteristics of macrosegregation in NbTi alloy.

**Figure 9:**
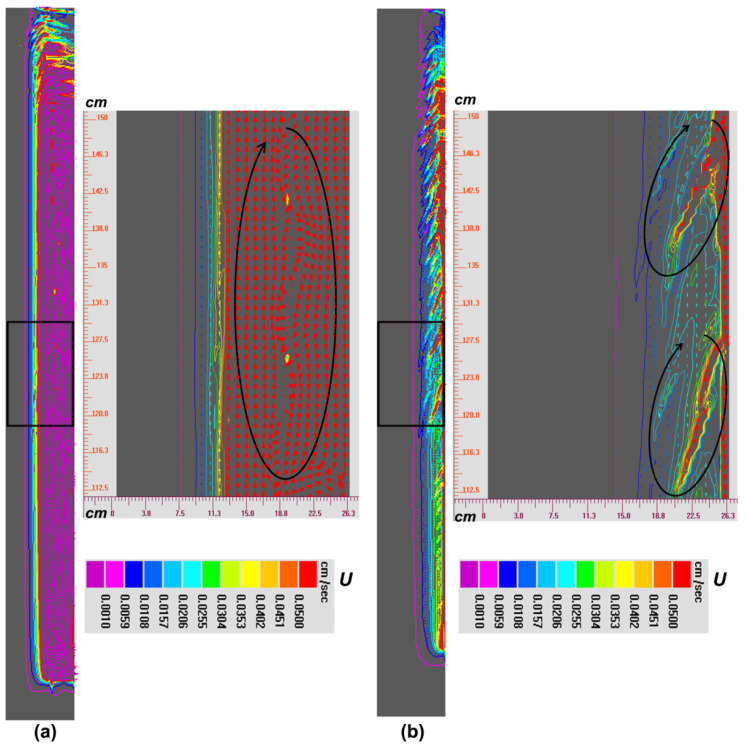
The distribution and evolution of the flow field during the early (**a**) and middle (**b**) stages of solidification in the NbTi ingot. (**a**) 1000 s; (**b**) 2700 s. The enlarged views of the flow field in the black box are also attached, respectively. The flow circulation is indicated by the black arrow.

At the beginning of solidification, due to the strong cooling effect of the mold, heat can be transferred outward quickly and the temperature near the mold/melt interface drops dramatically, consequently forming the huge temperature gradient. Therefore, the cool interdendritic liquid flows downward near the side mold owing to its heavy density, and then it turns upward along the centerline considering its high temperature and light density. As solidification proceeds, the solidified layer gradually increases and its thermal conductively becomes weaker, and hence there forms the thick thermo boundary layer in the front of solidification. In such case, the downward driving force driven by the temperature gradient decreases. At the same time, with the solute being expelled from the solid to the interdendritic melt, there exists the obvious enrichment of solute in the mushy zone, which can drive the ascending flow. When the solute continues to accumulate, namely ~1000 s, the solutal effect begins to dominate the downward thermal effect, and hence the clockwise current forms, as shown in Figure 9a. Such a flow mode can last until the end of solidification (Figure 9b), which causes the final positive segregation in the top and the negative segregation in the bottom. However, in the late solidification, the single systemic circulation gradually branches indicting the instability of the flow as shown by two smaller flow circulations in Figure 8b, which can trigger the occurrence of CS as discussed below. 

**Figure 10:**
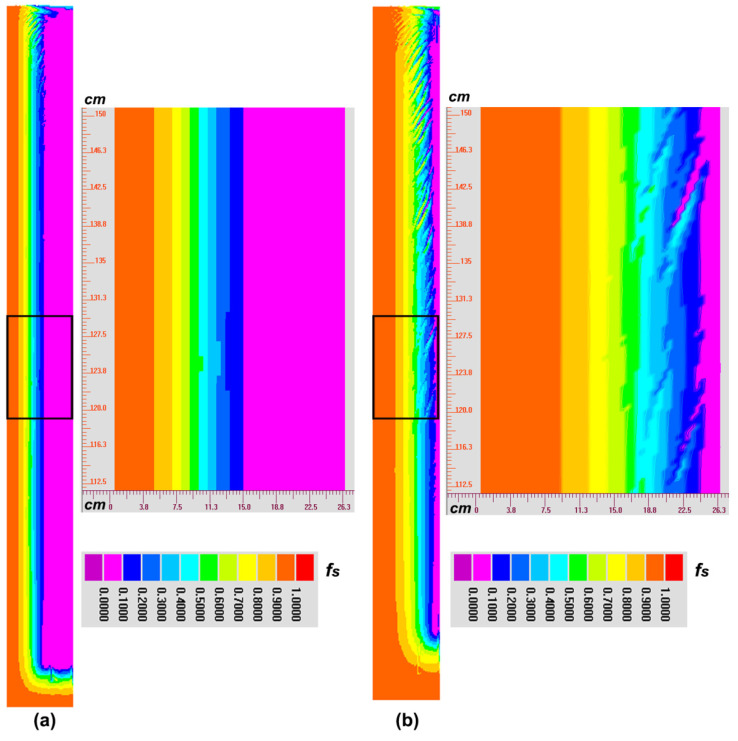
The distribution and evolution of isolines of solid fraction during the early (**a**) and middle (**b**) stages of solidification in the NbTi ingot. (**a**) 1000 s; (**b**) 2700 s. The enlarged views of solid fraction isolines in the black box are also attached, respectively.

To illustrate the formation mechanism and conditions of CS, the isolines of solid fraction are further extracted in Figure 10. At the initial stage of solidification (1000 s), although the solutal effect governs the flow field, but the solutal enrichment is not sufficiently obvious and at that moment the opposite thermal effect cannot be neglected, which causes the weak thermo-solutal convection in the mushy zone and the sequential solidification still exists, as shown in Figure 9a. With the development of solidification (2700 s), the solute continues to accumulate in the mushy zone and the ascending natural convection strength increases until the local mush destabilization occurs. When the initial micro channel is triggered, its main manifestations are the distortion of the solidification front and the wavy distribution of solid fraction isolines (Figure 10b) rather than the straight type appeared in Figure 10a. 

In addition, further calculations find that, when the angle between the interdendritic flow velocity and temperature gradient is smaller than 90°, the destabilization phenomenon of the mushy zone can last. It is because in such a situation, the increment rate of the solid fraction becomes slower and even negative (indicating the remelting of the solidified zone) with the sudden increase in the flow velocity, both of which can cause smaller local flow resistance and bigger permeability compared with the surrounding regions. According to hydromechanics, melt always flows along the low-resistance direction, and hence, in the interior of channel, the solute-enriched melt flows towards the growth direction of the channel, which can be seen by comparing the flow field, solid fraction isolines and concentration distributions (Figure 8, Figure 9 and Figure 10b). That is, the increase in local flow can be strengthened, and hence the flow instability and mush destabilization can be maintained rather than fade away. It should be stressed that the formation of CS can be divided into two stages: channel initiation and growth. The initiation stage is driven by strong natural convection and flow instability, which can be disturbed by either thermal or solutal fluctuation. For instance, the increase in solute or the decrease in temperature can both strengthen the local flow. When the local solidification is suppressed, it in turn accelerates the flow velocity and, hence, the destabilization of the flow field and mushy zone can survive, and the micro channel can continue to grow up into the macro channel in the completely solidified ingot. 

### 5.2. Effect of Cooling Rate on the Final Macrosegregation in NbTi Alloy 

**Figure 11:**
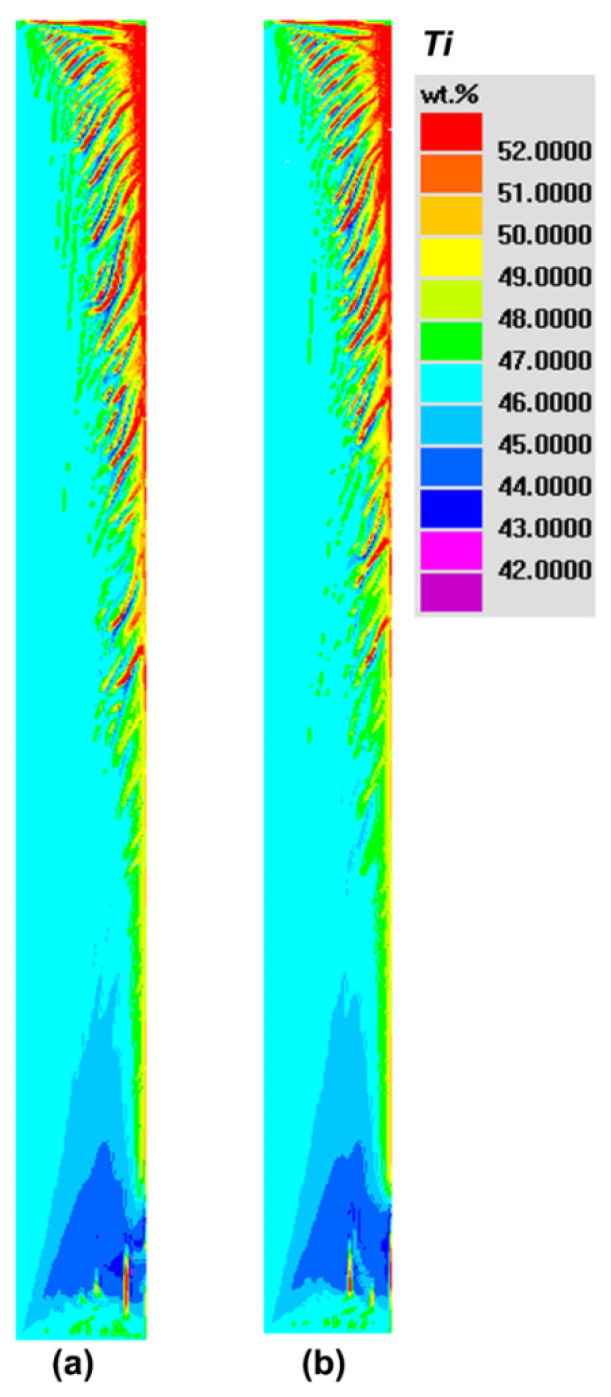
The effect of cooling rate on the final macrosegregation of Ti. The interface heat-transfer coefficients between mold and melt are increased to be 2500 (**a**) and 3000 W m^−2^ K^−1^ (**b**), respectively.

Based on the above simulation results, the origin of CS is driven by sufficiently strong flow during solidification. Hence, it can be predicted that the strong cooling effect can reduce the CS and the global macrosegregation in engineering. To reveal the effect of the different cooling methods on the final macrosegregation including CS, a series of simulation cases with different interface heat-transfer coefficients of mold/melt were carried out. Figure 11 illustrates the final distributions of Ti segregation when the heat-transfer coefficient is increased from 2000 to 2500 and 3000 W m^−2^ K^−1^, respectively. By comparing Figure 8c and Figure 11, there are still severe channel segregations in the cases with the faster solidification rate, which is consistent with the above experimental results in Figure 1. However, in terms of the number and severity of channels, both of them appear to be decreasing tendency when cooling rate increases. 

Table 3 lists the quantitative statistic results of convection strength and segregation extent in different cooling conditions. It can be found that, with the increase in cooling rate, the interdendritic velocity induced by thermal and solutal gradients is weakened during solidification, as are the average and maximum flow velocities in the whole ingot (0.1 < *f*_s_ < 0.7). When the flow strength is decreased, the transport of solute in the macro scale is hindered to some extent, and, hence, the maximum positive and global macrosegregation is reduced in the final ingot. In addition, through the formation mechanism of CS mentioned above, the risk of flow instability can be also lowered when the flow velocity becomes smaller. For instance, the maximum Ti content in the channel, and the channel length and number are all brought down with the increase in the interface heat-transfer coefficient. Thus, both simulations and experiments validate the reasonability of reducing the macrosegregation including channel segregation by increasing the cooling capacity of the mold in engineering. 

### 5.3. Rayleigh Number Criterion of CS Initiation in NbTi Alloy 

**Figure 12:**
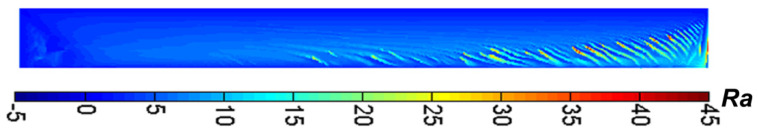
The Rayleigh number distribution in NbTi alloy.

Besides the formation mechanism and effecting factors of CS, its prediction criterion and model have widely attracted attention from researchers and engineers. Currently, there mainly exist two prediction models including the Suzuki and Rayleigh number (*Ra*) [22]. In the former, only the effect of thermal parameters is considered, and hence the critical Suzuki number varies with different alloy systems. In contrast, *Ra* stands for the ratio between the thermo-solutal driving force and the dragging force induced by melt viscosity, and it not only includes the temperature parameters but also comprises the microstructure, permeability and dynamic viscosity. Hence, it can be used to compare the difficulty of CS formation in various alloys, and has been widely used in SnPb-, GaIn-, and Ni-base superalloys and steels [26,27,28,29,30,31,32,33]. In this study, to directly extract the physical parameters used in *Ra* expression, it was further rewritten as follows.

(7) $$Ra=\frac{\Delta \rho }{\rho_{0}}\frac{g\bar{K}}{R\mu_{l}}=[\beta_{C}(C_{l}-C_{ref})+\beta_{T}(T-T_{0})]\frac{g{\left(1-f_{s}\right)}^{3}d_{s}^{2}G}{180\mu_{l}f_{s}^{2}\varepsilon }$$

where *R*, *G*, *ε* are solidification speed, temperature gradient and cooling rate, respectively. In Equation (7), the solid fraction *f*_s_ is selected on average as 0.15, in which it is always considered as the onset site of initiate channel segregation [31]. The distribution contour of *Ra* in the air-cooled ingot is depicted in Figure 12. It shows that, with the increase in distance from the side surface, the Rayleigh number increases first and then decreases. As mentioned above, the flow direction will be quickly overturned when solutal gradient governs the opposite temperature gradient with the development of solidification. Owing to the solute redistribution and accumulation of segregation, the density inversion becomes larger and, correspondingly, *Ra* also increases according to Equation (7). However, at the end of the solidification, the simultaneous solidification phenomenon occurs, and hence fast solidification lowers the Rayleigh number although the density contrast between the interdendritic liquid and bulk melt is large. 

By further comparing the *Ra* distribution in Figure 12 and the final CS positions in Figure 8c, we find that there is a critical Rayleigh number of 15 at the onset site of CS. When Rayleigh number is smaller than the critical value, CS disappears. Coincidentally, the critical criterion is located in the range of 9–25 proposed by Rad in industrial steel ingots [31]. It is worth noting that the current *Ra* criterion is a single value, which can provide the more accurate prediction of CS sites and guide the optimization of the solidification process in practice compared with the critical range. In the current simulations, the positions with the locally maximum Rayleigh number agrees well with the real onset sites of CS, and such self-consistency sufficiently proves the accuracy of the suggested *Ra* criterion and the macrosegregation model. In addition, according to the experimental result in Figure 1, there are eight main channels in the top test piece, which is generally consistent with the predicted number of CS by the *Ra* criterion. Meanwhile, the onset sites of CS from the simulation and *Ra* criterion are located in 1/4–3/4 radius of the ingot, which is also successfully validated by the experimental results. Therefore, the proposed *Ra* criterion provides a high-efficient and significant tool to forecast the initiation of CS in NbTi alloy, and the process parameters during solidification can be optimized to reduce CS in engineering. 

## 6. Conclusions

In this study, via the numerical simulations and experimental characterizations, the distribution characteristics, evolution process, potential mechanism and affecting factor in Nb-47 wt.% Ti alloy are elucidated in detail, and the main conclusions are summarized below.

1. By comparing the macrostructure, grain size, compositional segregation and 3D details of channel segregation in He- and air-cooled ingots, it can be found that severer segregation in the air-cooled ingot is caused by coarser grains and the resulting stronger natural convection during solidification. After increasing cooling by He treatment, it reduces the interdendritic flow velocity, consequently lowering the positive, global and channel-type segregations. The key effect of the cooling capacity of the mold on macrosegregation including CS was sufficiently proven by the numerous simulations.
2. Macrosegregation simulations show that the formation of CS in NbTi alloy includes two stages of channel initiation and growth. CS initiation is triggered by the flow instability and mush destabilization caused by the large density contrast and the strong thermo-solutal convection. Under the interaction of solidification and flow, the micro channel can continue to grow into a macro channel.
3. Based on the Rayleigh number distribution and the final CS sites, the critical *Ra* criterion of 15 is successfully proposed in NbTi alloy for the first time. When the Rayleigh number is lower than the critical value, CS disappears. It provides a significant tool to predict CS and fabricate the homogenized NbTi ingot in engineering.

In summary, although the above conclusions are obtained from the current Nb-47 wt.%Ti alloy, they are still prospectively valid for other NbTi alloys with different metal composition percentages. On one hand, the composition percentages in NbTi alloy can induce the different density inversions and the strength difference of natural convection during solidification. Generally, with the increase in the initial Ti content, its segregation becomes more severe. However, in terms of the stronger cooling rate, it still weakens the interdendritic flow, refines the grain size and consequently lowers the positive and global extents of macrosegregation in various NbTi alloys. On the other hand, it has been widely considered that *Ra* can be used to compare the sensitivity of CS in common alloy systems, and hence the proposed critical value is applicable to optimize the solidification process to reduce CS. When the original content of Ti is lowered, *Ra* will decrease considering its smaller density difference between the mushy zone and bulk melt, and therefore, in these low-Ti alloys, CS can be inhibited to some extent.

## Figures and Tables

**Table 1:** The main thermo–physical parameters used in the current simulations.

Parameter	Symbol	Value
Solutal expansion coefficient	*β_C_*	0.8 × 10^−2^ (wt.%)^−1^
Thermal expansion coefficient	*β_T_*	1.87 × 10^−5^ K^−1^
Liquidus slope	*m_l_*	−11.4 K (wt.%)^−1^
Secondary dendritic arm spacing	*d_s_*	200 μm
Dynamic viscosity	*μ_l_*	0.0042 Pa·s
Specific heat capacity	*c_p_*	2000 J kg^−1^ K^−1^
Latent heat	∆*H*	150,000 J kg^−1^
Heat conductivity	*λ*	30 W m^−1^ K^−1^
Density	*ρ*	5460 kg m^−3^
Melting point of pure Nb	*T_m_*	2750.15 K
Equivalent partition coefficient	*k*	0.75

**Table 2:** The boundary conditions used in the simulations.

	*ρ*, kg m^−3^	*C_p_*, J kg^−1^ K^−1^	*λ*, W m^−1^ K^−1^	*T_0_*, K
Mold	8900	385	380	323.15
Interface heat-transfer coefficient *h*_i_, W m^−2^ K^−1^		Liquid–top surface: 50 Mold–top surface: 50 Liquid–mold: 2000 Mold–air: 200 + 4 × 5.67 × 10^−8^ × 0.9 × *T*^3^		

**Table 3:** The interdendritic flow velocity and segregation extent during solidification with different cooling rates.

Interface Heat-Transfer Coefficient, W m^−2^ K^−1^	Average Interdendritic Flow Velocity ^1^, mm/s	Max. Interdendritic Flow Velocity ^1^, mm/s	Max. Ti Content ^2^, wt.%	GM ^2^	Max. Ti Content in CS ^2^, wt.%	Max. Length of CS, mm^2^	Number of CS ^2^
2000	0.105	0.944	63.66	0.0412	58.49	19.76	24
2500	0.104	0.924	63.39	0.0406	58.31	17.89	21
3000	0.103	0.794	63.29	0.0397	58.17	15.81	20

Note: ^1^ means the solidification time of 2700 s in which the solidification proceeds towards half of the whole ingot, and ^2^ denotes the final solidification time in three cases.

## Data Availability

The data presented in this study are available on request from the corresponding author.
